# Severe paravalvular leakage caused by delayed dislocation of valve prosthesis after transcatheter aortic valve replacement for rheumatic aortic valve disease: A case report

**DOI:** 10.1097/MD.0000000000049867

**Published:** 2026-08-21

**Authors:** Chengyi Xu, Hui Guo, Xi Su

**Affiliations:** a Department of Cardiology, Wuhan Asia Heart Hospital, Wuhan, Hubei, China.

**Keywords:** aortic stenosis, case report, paravalvular leakage, transcatheter aortic valve replacement, valve dislocation, valve-in-valve

## Abstract

**Rationale:**

Among patients undergoing transcatheter aortic valve replacement (TAVR), serious perivalvular leakage (PVL) caused by valve dislocation is a rare but serious complication.

**Patient concerns:**

A 50-year-old male patient was admitted due to chest tightness.

**Diagnosis:**

Patient was diagnosed with rheumatic valvular heart disease, aortic stenosis, severe acute insufficiency, acute decompensated heart failure, cardiac function grade IV (New York Heart Association), and multiple organ failure.

**Interventions:**

After TAVR, delayed left ventricular dislocation of the valve prosthesis caused severe PVL and severe heart failure. A 2nd emergency TAVR operation was performed, and the TAVR artificial valve was implanted again using the transcatheter “valve-in-valve” technique.

**Outcomes:**

Postoperative evaluation showed mild PVL, and the clinical symptoms were relieved.

**Conclusions:**

Rheumatic aortic valve disease and multiple organ failure, severe PVL caused by delayed dislocation of valve prosthesis after TAVR is rare, which might necessitate prosthetic valve reimplantation using the transcatheter “valve-in-valve” technique.

## 1. Introduction

Aortic stenosis (AS) is a narrowing of the aortic valve caused by atherosclerotic deposits and consequent calcification, rheumatic heart disease, or congenital anomalies.^[1]^ The prevalence of AS is about 0.6% to 0.9% in the United States.^[1]^ Severe symptomatic AS is associated with a poor prognosis and a 25% annual mortality without aortic valve replacement.^[2]^ Transcatheter aortic valve replacement (TAVR) is the preferred treatment for older adults with symptomatic AS.^[3]^ Still, TAVR can lead to complications like severe paravalvular leakage (PVL) due to valve dislocation, which is an extremely serious complication of TAVR.^[4]^ This study reports a patient with rheumatic valvular heart disease, moderate aortic stenosis, severe acute insufficiency, acute decompensated heart failure, New York Heart Association functional class IV, and multiple organ failure who underwent TAVR and developed severe PVL caused by delayed dislocation of the valve prosthesis.

## 2. Case report

This study was approved by the Medical Ethics Committee of Wuhan Asian Heart Hospital (2023-B014). Informed consent was obtained from the patient.

A 50-year-old male was admitted to Wuhan Asian Heart Hospital on October 5, 2021, due to chest tightness for 2 weeks. Over the past 6 months, the patient had felt chest tightness and shortness of breath during activities. In March 2021, the patient was diagnosed with rheumatic heart disease and hospitalized at a local hospital due to symptom aggravation. Transthoracic echocardiography (TTE) revealed rheumatic heart disease, severe AS, and severe aortic valve insufficiency. The patient was treated symptomatically but without improvement.

Physical examination revealed tachypnea (>20 breaths/min), emaciation, jaundice, rhonchi, moist lung rales, abdominal rebound pain, and slight lower limb edema. The patient had a grade 3/6 systolic blowing murmur transmitted to the neck that could be heard in the 1st and 2nd auscultation areas of the aortic valve. Moderate sigh-like murmurs were heard in diastole. Laboratory examination revealed severe renal and liver abnormalities: alanine aminotransferase 939 IU/L (reference: 0–50 IU/L), aspartate aminotransferase 1064 IU/L (reference: 0–50 IU/L), total bilirubin 108.6 μmol/L (reference: 5–21 μmol/L), direct bilirubin 56.5 μmol/L (reference: 0–3.4 μmol/L), serum creatinine 193 μmol/L (reference: 57–97 μmol/L), and N-terminal B-type natriuretic peptide precursor >35,000 pg/mL. An electrocardiogram showed sinus tachycardia. TTE showed AS and severe regurgitation (peak velocity of forward flow through the aortic valve was 3.8 m/s, the average peak velocity was 2.6 m/s, the peak pressure difference was 58 mm Hg, and the mean aortic valve gradient was 31 mm Hg) (Fig. 2B), moderate mitral regurgitation, severe left ventricular dilatation (including left ventricular end-diastolic/systolic diameter: 85 mm/79 mm), and left ventricular ejection fraction of 17%.

**Figure 1.**
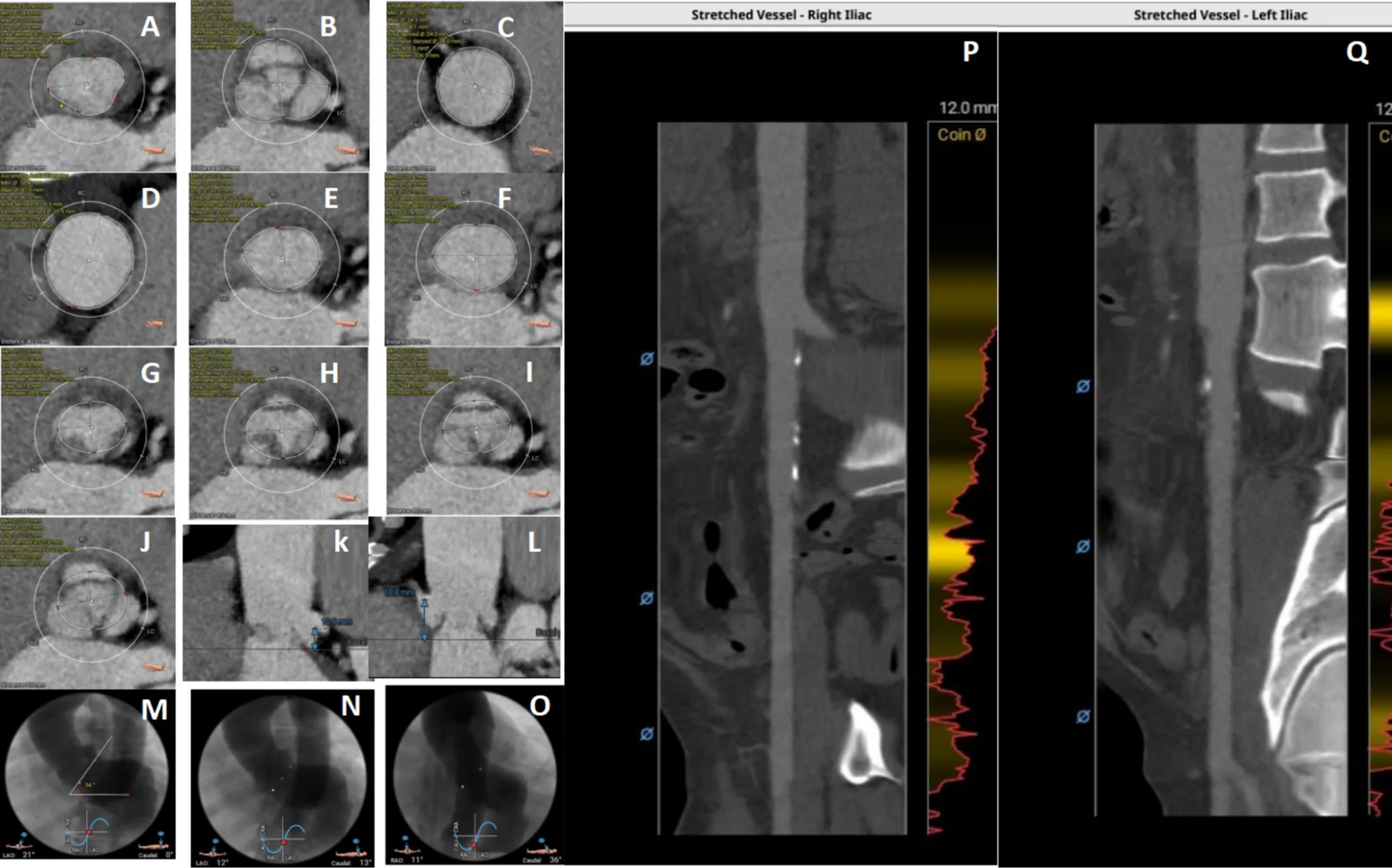
Preoperative 3-dimensional computed tomography (CT) evaluation of aortic root structure, virtual valve annulus angulation, cross-valve, valve release position, and transcatheter aortic valve replacement (TAVR) surgery path. The inner diameters of the aortic valve virtual annulus (A), sinus (B), sinus junction (C), and ascending aorta (D) were 29.8, 38.8, 34.0, and 39.9 mm, respectively. The inner diameters of the left ventricular outflow tract 2.5 and 5 mm below the plane of the virtual annulus (E and F) were 30.4 and 32.5 mm, respectively. The maximum inner diameters of supravalvular structures 2 (G), 4 (H), 6 (I), and 8 mm (J) above the plane of the virtual annulus were 27.2, 27.8, 27.2, and 28.0 mm, respectively. The height of the left coronary artery was 10.6 mm (K). The height of the right coronary artery was 18.8 mm (L). The results showed that the angle of the virtual valve ring was 54°, close to the standard of the transverse position (M). Middle position of the right coronary sinus (cross-valve position): left anterior oblique 12°, foot position 13° (N). Left and right coronary sinus overlapping position (valve release position): right anterior oblique 11°, foot position 36° (O). The right (P) and left (Q) femoral arteries had no obvious tortuosity or calcification, and the vessel diameter was >6.0 mm.

The patient was diagnosed with rheumatic valvular heart disease, AS, severe insufficiency complicated with acute decompensated heart failure, New York Heart Association functional class IV, and multiorgan failure. After a multidisciplinary discussion, TAVR was recommended. Preoperative 3-dimensional computed tomography angiography was performed to evaluate the aortic root structure, virtual valve annulus angulation, cross-valve, valve release position, and TAVR surgery path before the operation (Fig. 1).

**Figure 2.**
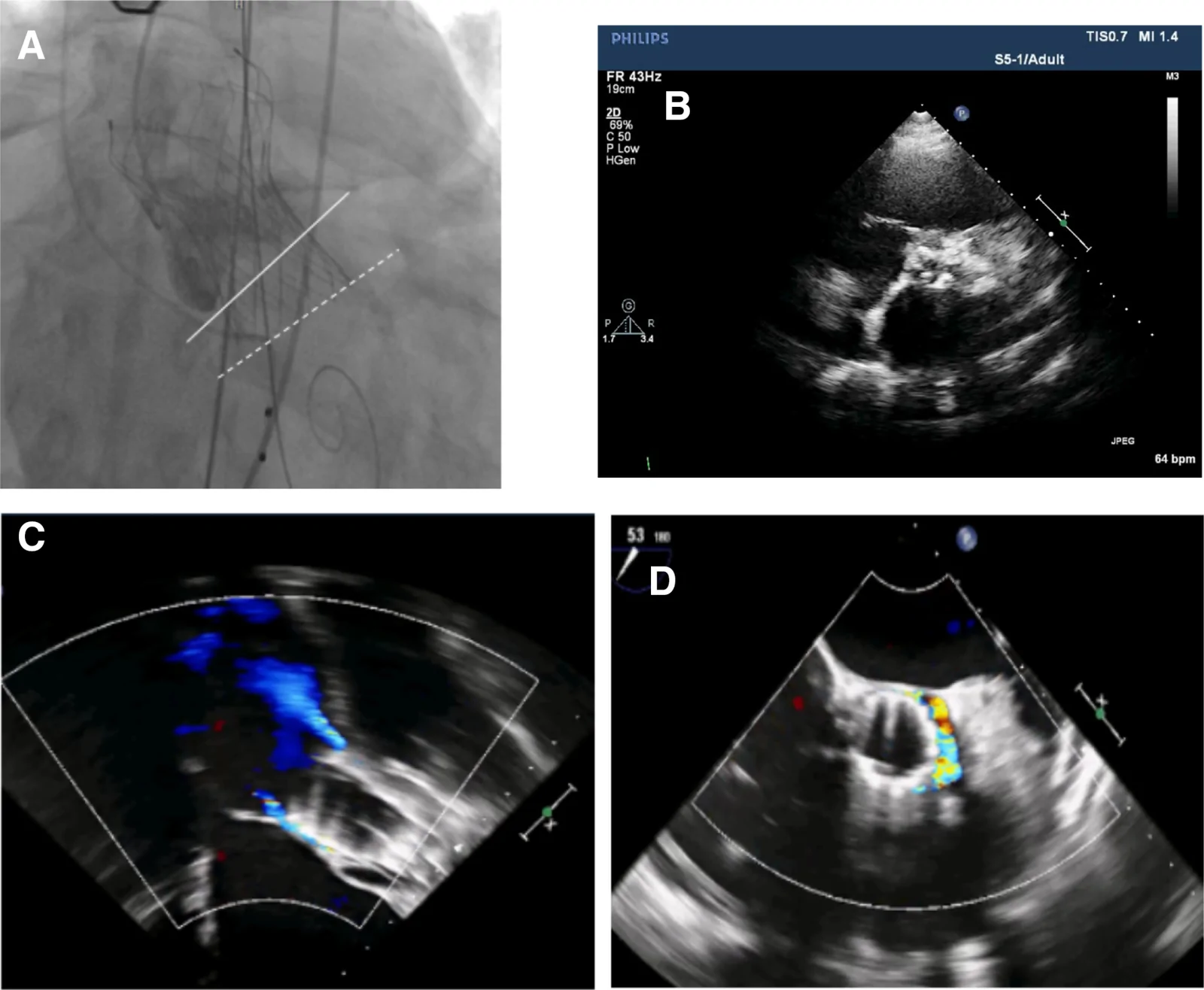
Digital subtraction angiography (DSA), transesophageal echocardiography (TEE), and transthoracic echocardiography (TTE) evaluation during the 1st transcatheter aortic valve replacement (TAVR) operation. The solid white line shows the plane of the valve ring, and the white dotted line shows the plane of the bottom of the valve prosthesis (A). Preoperative TTE evaluation showing severe aortic stenosis and regurgitation (B). The postoperative TEE evaluation results showed mild and moderate perivalvular leakage (PVL) (C) in the long-axis plane and 2 mm reflux beam (D) in the 1 and 5 o’clock directions in the short-axis plane.

TAVR was performed under general anesthesia on the 3rd day of admission via the right femoral artery using a 22-Fr Gore vascular sheath. A Vitaflow Ⅱ TAV30 valve stent (Shanghai Minimally Invasive Medical Device Co.) was preinstalled, and the valve was directly released after positioning, without balloon pre-expansion. The aortic valve circumference was 93.6 mm, and the mean annular diameter was 29.8 mm. For patients with AS, the oversize rate must be 10% to 15%, as per the Chinese guidelines.^[5]^ The valve was selected according to Yao et al.^[6]^ The valve was released at the plane of the virtual valve ring (zero position), and the left ventricle was significantly dislocated. After partial recovery of the valve, it was successfully released again 2 mm above the virtual valve ring plane. During the operation, the valve prosthesis extended 8 mm below the plane of the valve. Transesophageal echocardiography showed mild-to-moderate PVL (Fig. 2), and the operation was completed.

**Figure 3.**
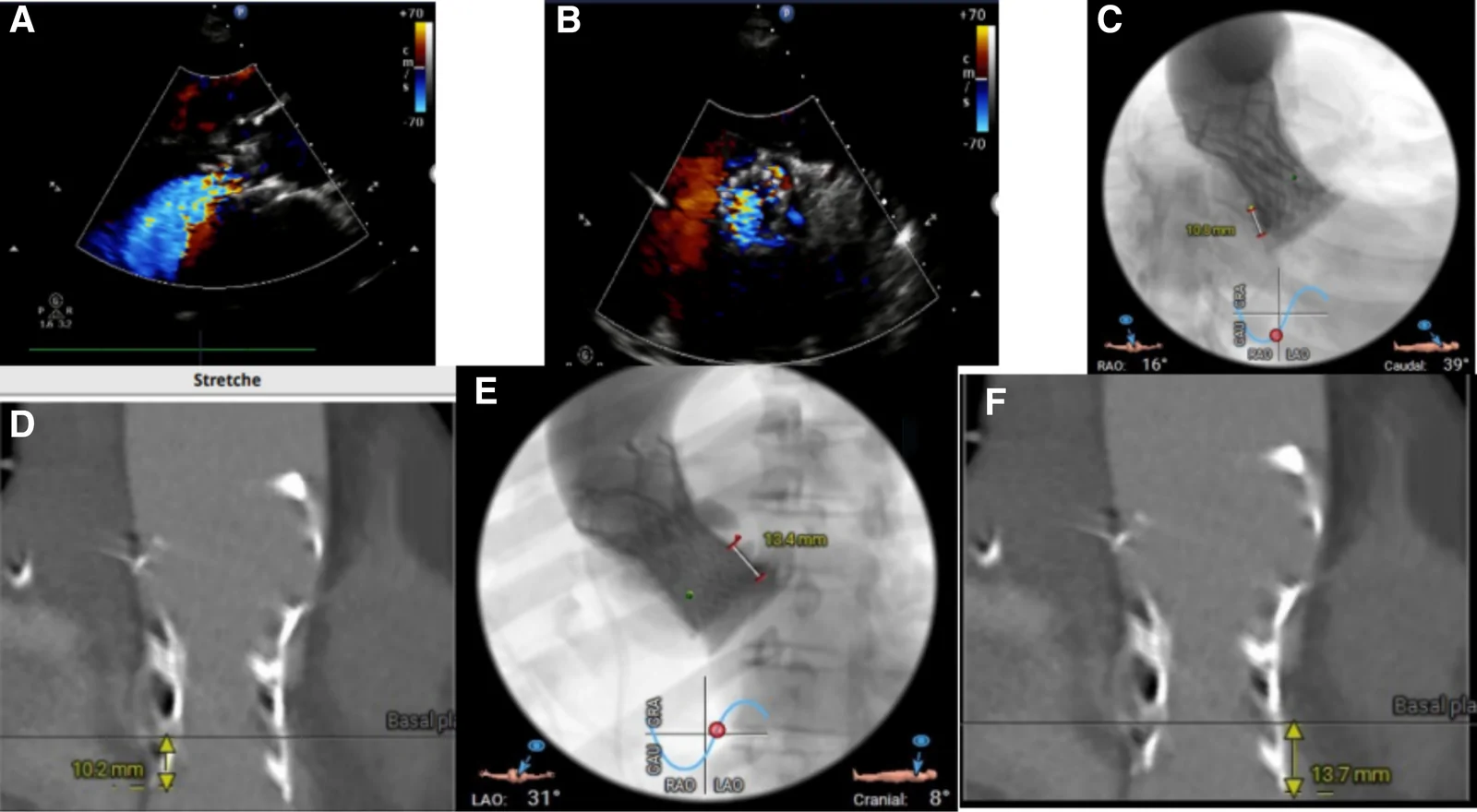
Bedside transthoracic echocardiography (TTE) evaluation and the relationship between the aortic root complications and the position of the bottom edge of the valve prosthesis and the virtual valve ring evaluated by 3-dimensional computed tomography (CT) before the 2nd transcatheter aortic valve replacement (TAVR) operation. The evaluation results of the long axis (A) and short axis (B) of the bedside TTE showed moderate and severe central regurgitation of the aortic valve and severe perivalvular leakage (PVL). At the tangent position of the valve, CT was used to measure the distance from the bottom of the valve prosthesis to the plane of the valve ring; the results showed 10 to 11 mm (C and D) on the side of the non-coronary sinus and 13 to 14 mm (E and F) on the opposite side.

Transient III° atrioventricular block occurred during TAVR. Tracheal intubation was removed 2 hours after the operation, and the arrhythmia recovered. The temporary pacing electrode was removed 24 hours after the operation. The patient’s condition improved after the operation. Four days after surgery, the patient suffered from sudden dyspnea and acute left heart failure. Bedside TTE results showed moderate-to-severe central regurgitation and severe PVL (Fig. 3A and B). Emergency tracheal intubation and invasive ventilator treatment were performed. Computed tomography angiography showed that the valve prosthesis was significantly dislocated to the left ventricle at the left coronary sinus side; no vascular complications at the aortic root were found (Fig. 3C–F). The patient was in a state of multiple organ failure, and open surgery was contraindicated. After discussion, it was suggested that the 2nd emergency TAVR should be performed using the “valve-in-valve” (ViV) technique. The Vitaflow Ⅱ TAV30 valve stent was implanted again at the valve ring plane.

**Figure 4.**
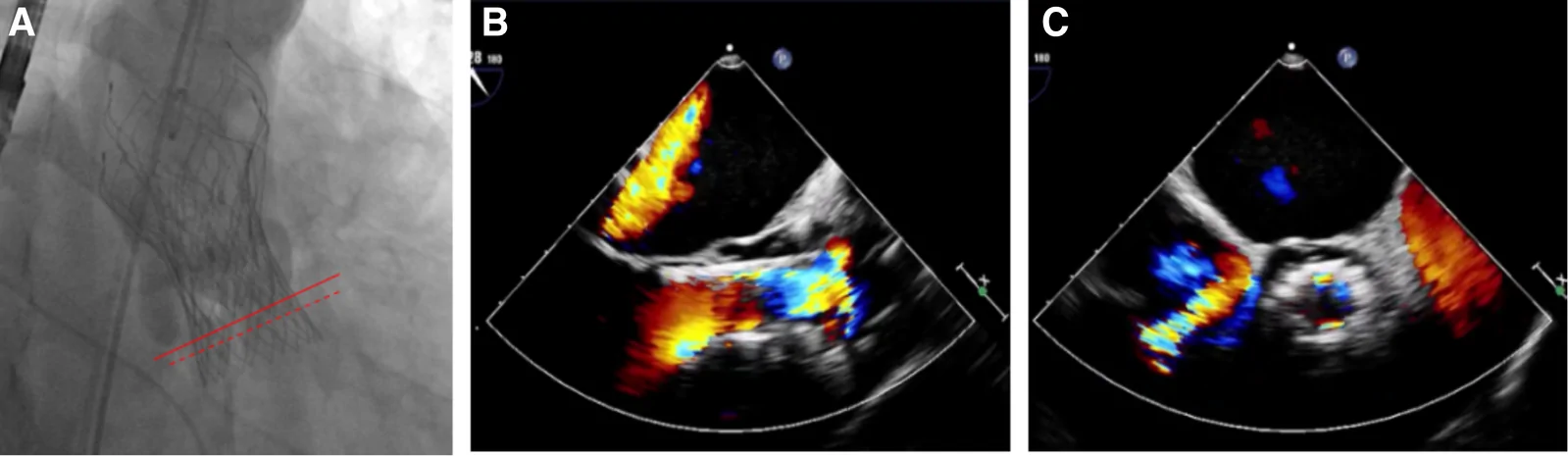
Postoperative digital subtraction angiography (DSA) and transesophageal echocardiography (TEE) evaluation. Digital subtraction angiography (DSA) and transesophageal echocardiography (TEE) evaluation. (A) The solid red line shows the plane of the valve ring, and the red dotted line shows the plane of the bottom of the 2nd valve prosthesis. (B and C) The TEE evaluation results after the 2nd TAVR operation showed that there was no PVL and central reflux in the long-axis plane and no reflux beam in the short-axis plane. PVL = paravalvular leakage, TAVR = transcatheter aortic valve replacement.

Postoperative digital subtraction angiography and transesophageal echocardiography results were good, and no obvious PVL was found (Fig. 4). The patient was discharged on the 6th day after the 2nd TAVR. There were no clinical symptoms or signs of cardiac failure after 7 months of outpatient follow-up.

## 3. Discussion

This study reported a case of severe PVL caused by valve prosthesis delayed dislocation after TAVR for rheumatic aortic valve disease, followed by a 2nd emergency TAVR operation using the transcatheter ViV technique. This case report may provide clues for managing PVL caused by valve prosthesis dislocation after TAVR.

Previous series have shown that transcatheter valve embolization and migration are uncommon but high‑risk complications, occurring in approximately 1% of TAVR procedures and usually within the periprocedural or very early postprocedural period.^[7–11]^ Most reported cases of retrograde migration into the left ventricular outflow tract have involved degenerative calcific aortic stenosis, frequently related to low initial positioning, undersizing, or suboptimal valve expansion, and many have required surgical rescue or percutaneous repositioning techniques rather than repeat TAVR. In contrast, our patient had rheumatic aortic valve disease with severe biventricular dysfunction and multiorgan failure, developed delayed ventricular migration a few days after TAVR with resultant severe PVL, and was successfully treated by a 2nd “valve‑in‑valve” procedure without conversion to open surgery, highlighting that this strategy can be feasible even in critically ill patients when surgical risk is prohibitive.

Nevertheless, for emergency TAVR, mild-to-moderate or moderate PVL is acceptable, especially in critically ill patients. Due to limited experience at our center, the results of the 1st TAVR were suboptimal, which may have contributed to the delayed valve dislocation. At the study hospital, in patients with aortic stenosis, the incidence of PVL above a mild level is about 25%, while the incidence of severe perivalvular leakage does not exceed 5%. The Vitaflow II TAV valve stent is the most commonly used valve for TAVR at our center. The historical total complication rate is about 7%, similar to the rates reported in the literature. Indeed, in multicenter studies and registry analyses, the 30-day overall mortality for Vitaflow II has been reported as 2.7% to 7.6%, stroke rates as 2.4% to 3.2%, major vascular complications as 5.5% to 7.2%, and permanent pacemaker implantation rates as 9.1% to 19.1%.^[12,13]^

PVL is the most common complication after TAVR and is usually caused by calcification, valve eccentricity, or a small prosthesis size. Symptomatic patients require reintervention.^[12]^ Severe or extremely severe PVL caused by severe dislocation can be dangerous and requires urgent intervention.^[7]^ Valve displacement is related to the underlying cause of valvular disease, the anatomical structure of the aortic root, the degree and distribution of valve calcification, the operator’s skills, and several other reasons.^[14]^ Although the valve prosthesis size was selected according to guidelines and prosthesis characteristics, it may have been undersized, leading to displacement. The exact cause of valve dislocation will remain unknown because there is no way to observe in real-time what occurs after valve deployment or to determine the exact pathophysiological condition of the implantation site. Nevertheless, it can be hypothesized that rheumatic changes of the valve, that is, stenosis due to myxoid changes, degenerative changes in the anchorage of the valve, and poor anchoring of calcified lesions, can contribute to valve displacement. On the other hand, it was observed that the valve was in a low initial position, which is probably a major reason for the dislocation.

Among strategies for managing delayed valve dislocation or embolization, the transcatheter ViV approach has emerged as a viable rescue technique to secure the dislodged valve and restore hemodynamics, avoiding high-risk open-heart surgery.^[15]^ Observational data, such as the CENTER study, confirm that ViV–transcatheter aortic valve implantation is a safe and effective treatment with 30-day and 1-year mortality and stroke rates comparable to native valve transcatheter aortic valve implantation.^[16]^ The valve prosthesis is reimplanted to replace the original prosthesis, thus preventing the displacement of the 2 valves.^[8]^ When patients’ symptoms after TAVR surgery worsen, or TTE indicates poor hemodynamics, delayed valve dislocation should be highly suspected, and emergency treatment should be considered.^[17]^ In the case reported here, the initial use of a self-expanding valve (SEV) without balloon pre-dilation in a rheumatic lesion lacking severe calcification may have resulted in insufficient radial support, contributing to delayed dislocation. For the emergency ViV intervention, the choice between a balloon-expandable valve and an SEV is critical. While a balloon-expandable valve might offer specific anchoring advantages, a recent meta-analysis^[18]^ and the LYTEN trial^[19]^ demonstrated that SEVs provide superior hemodynamic profiles, including larger effective orifice areas and lower transaortic gradients, in ViV procedures for failed prostheses. Therefore, deploying a 2nd SEV was a hemodynamically sound strategy for this rescue procedure.

Regarding central aortic insufficiency, after the valve shifted to the left ventricle, severe PVL occurred. Left ventricular dislocation of the transcatheter valve can result in loss of valve function, accompanied by severe valve regurgitation and PVL. Especially, severe PVL may interfere with the true source of the aortic regurgitant flow. In addition, severe PVL manifested as severe aortic insufficiency or regurgitation on the long axial section of the aortic valve on the Doppler examination, and it manifested as central regurgitation on the ultrasound. Therefore, it was essentially PVL.

Releasing the valve 2 mm above the virtual valve ring plane may pose a high risk of aortic embolization. The 1st release occurred at the valvular ring plane, and the valve slipped significantly. The 2nd release occurred 2 mm above the ring plane of the virtual valvular ring plane. Considering the structural limitations of the valvular ring plane, the valve was released to the left ventricle to leave some space, and the final release was successful. Of course, there is a risk of aortic valve dislocation with a high release, which is why we considered using a retrievable valve and tried different release depths during surgery.

After discharge, the patient was followed in a dedicated valve clinic with clinical assessment, electrocardiogram, and transthoracic echocardiography at 1, 3, and 6 months and annually thereafter, together with guideline‑directed heart failure therapy and standard post‑TAVR antithrombotic management, and he remained free of heart failure symptoms during 7‑month follow‑up.

In conclusion, severe PVL caused by delayed dislocation of the valve prosthesis after TAVR is rare and may necessitate prosthetic valve reimplantation using the transcatheter ViV technique.

## Acknowledgments

This study was supported by the Youth Project of the Wuhan Municipal Health Commission (key project) (WX19Q13).

## Author contributions

**Data curation:** Chengyi Xu, Hui Guo.

**Formal analysis:** Chengyi Xu, Hui Guo, Xi Su.

**Methodology:** Chengyi Xu, Hui Guo, Xi Su.

**Validation:** Xi Su.

**Writing – original draft:** Chengyi Xu.

**Writing – review & editing:** Hui Guo, Xi Su.
